# Metagenomic next-generation sequencing for the etiological diagnosis of rabies virus in cerebrospinal fluid

**DOI:** 10.3389/fmed.2023.982290

**Published:** 2023-02-09

**Authors:** Yong Liu, Xichao Mo, Ye Feng, Rodney E. Willoughby, Xing Weng, Yuyang Wang, Xing Li, Junling Gao, Jinfei Tian, Jie Peng

**Affiliations:** ^1^Intensive Care Unit, Shenzhen Hospital, Southern Medical University, Shenzhen, Guangdong, China; ^2^Department of Infectious Diseases, Nanfang Hospital of Southern Medical University, Guangzhou, Guangdong, China; ^3^Changchun Veterinary Research Institute, Chinese Academy of Agricultural Sciences, Changchun, Jilin, China; ^4^Department of Pediatrics, Medical College of Wisconsin, Milwaukee, MI, United States; ^5^Department of Infectious Disease, BGI-Shenzhen, Shenzhen, Guangdong, China; ^6^Centre of Buddhists Studies, The University of Hong Kong, Hong Kong, Hong Kong SAR, China; ^7^Department of Medicine, LKS Medical Faculty, The University of Hong Kong, Hong Kong, Hong Kong SAR, China

**Keywords:** rabies, rabies virus, metagenomic next-generation sequencing, diagnosis, cerebrospinal fluid

## Abstract

**Background:**

Rabies is a highly fatal disease. Once symptoms develop, death usually occurs within days. Survivors were occasionally reported in the literatures. Ante-mortem diagnosis remains a challenge in most rabies endemic countries. A novel, accurate diagnostic assay is highly desirable.

**Methods:**

We used metagenomic next-generation sequencing (mNGS) to examine the cerebrospinal fluid (CSF) samples of a 49-year-old patient with rabies and validated the results by TaqMan PCR and RT-PCR/Sanger sequencing.

**Results:**

Metagenomic next-generation sequencing identified sequence reads uniquely aligned to the rabies virus (RABV). PCR confirmed the presence of the partial RABV N gene in the CSF. Phylogenetic analysis showed that the RABV grouped as an Asian clade, which is the most broadly distributed clade in China.

**Conclusion:**

Metagenomic next-generation sequencing may be a useful screening tool for the etiological diagnosis of rabies, especially in the absence of timely rabies laboratory testing or in patients with no exposure history.

## 1. Background

Rabies is a viral infection that has detrimental effects on the central nervous system. It can infect warm blooded mammals (1). The disease presents with specific symptoms of hypersalivation, difficulty swallowing, and hydrophobia in humans (2). Among all currently recognized infectious disease, rabies has the highest case-fatality rate, of nearly 100% (3, 4). Post-exposure prophylaxis (PEP) is effective (5), yet its supply and distribution systems are inadequate in many places and often very costly (6). Once symptoms develop, death usually occurs within days, even with intensive care (4, 7).

The laboratory diagnosis of rabies is imperative to guide public health measures like PEP and to strengthen awareness of rabies burden among policy-makers (8). However, ante-mortem diagnosis remains a challenge in most rabies endemic countries. The diagnosis of rabies is often clinical, sometimes leading to delayed, missed diagnosis or misdiagnosis, especially for infection without bites or paralytic rabies (3). Moreover, ante-mortem testing of all the pathogens causing encephalitis by conventional methods, such as smears, serologic tests, cultures, and pathogen-specific polymerase chain reaction (PCR), is impractical and resource consuming. The China Food and Drug Administration has not officially approved any commercial antibodies or molecular diagnostic kits for rabies diagnosis (9). Metagenomic next-generation sequencing (mNGS) has the potential to identify and genomically characterize infectious agents in a target-independent manner (10). Increasing evidence has shown that mNGS may play a role in identifying the etiological agent when conventional methods have failed (11).

Here, we report mNGS may be treated as a comprehensive, accurate, and efficient method for rapid and direct identification of rabies virus in the CSF, in case when we fail to come up with a definitive diagnosis of rabies by clinical characteristics or recommended conventional rabies laboratory diagnostic.

## 2. Materials and methods

### 2.1. Case summary

On 9 August 2017, a 49-year-old male, originally from the United States but resident in China for 10 years, sought help in Southern Medical University Shenzhen Hospital, Shenzhen. A stray dog had scratched him 1 week earlier and disappeared.

The patient was healthy until the stray dog scratched his left hand (August 1). The wound was about 1.0 cm in length, and was accompanied by minimal bleeding. Once cleaned and disinfected, the wound was dressed with gauze in a local clinic. The patient was not vaccinated for rabies at this time. Over the next 7 days, he developed paraesthesia and felt pain in his left arm and neck. He visited a local clinic complaining of difficulty swallowing and neck pain. He developed a low-grade fever (August 6), which kept rising. On the morning of August 8, the patient became agitated and apprehensive. He exhibited hyperventilation, aerophobia, hydrophobia, laryngeal, and diaphragmatic spasms. He was transferred to Southern Medical University Shenzhen hospital. The doctors in the Emergency Department made a clinical diagnosis of rabies, and a neurologist confirmed there were no other likely alternative diagnoses. He was admitted to the ICU at 10 p.m. on August 8.

This patient was once scratched by a stray dog when dealing with the dog 1 week before the onset. This suspicious dog went missing even with vigorous search. One of his adopted dogs, also with an unclear history of rabies vaccination, died several months earlier, possibly due to a fall. He had no history of vaccination against rabies. The patient denied any prior medical illness and he had a past history of heavy drinking.

On examination (8 August 2017), the patient was lucid and had a temperature of 38.5°C. He complained of persistent pain in his left arm and paraesthesia, difficulty in swallowing and mild dysarthria, but he was without spasm. He exhibited gaze nystagmus, ataxia of the left upper limb and trunk, decreased reflexes in the upper limbs, and bilateral extensor plantar responses. The only sensory abnormality was limited to the left upper limb. He showed normal cranial nerves functions, muscle strength (5/5) in the limbs, and muscle tone.

Laboratory investigations (12 August 2017) showed normal hemoglobin, coagulation, electrolytes, urinalysis, and electrocardiogram; however, C-reactive protein was increased to 25 mgL_1. There was a neutrophil leukocytosis and the plasma viscosity was increased by 1.83 mPa-s. Some linear opacification was found in the right lower lobe by chest X-ray. No bacterial pathogens were found by the culture of blood, urine, and stool.

Computed tomography imaging of the head (12 August 2017) found no abnormality. The patient showed neurological symptoms, suggesting the possibility of intracranial infection. However, evidences supporting the diagnosis of rabies was insufficient, as well as pathogens in nervous system were unclear. Based on these, we collected his CSF by lumbar puncture for etiological examination, as CSF samples are standard procedures for CNS infections. The CSF analysis did show mildly increased protein of 58.2 mg/dL. Culture of CSF was also negative. Although there was strong clinical suspicion, the diagnosis could not be confirmed as rabies test was not available in our institution. In fact, no commercial rabies test is licensed in China. PCR of rabies virus was provided by local CDC several days later only when clinical diagnosis reached consensus especially by infectious specialists.

Once the clinical diagnosis was made, tracheal intubation was performed to prevent aspiration. He received PEP including human rabies immunoglobulin (BPL, UK) (20 IU/kg, injected in the area in and around the wounds and the remaining volume injected intramuscularly at a site away from the site of vaccine administration) and human diploid cell rabies vaccine (Pasteur Mérieux, MSD Ltd.), ribavirin, Interferon-α. Deep sedation with ketamine and midazolam was also initiated. The bispectral index score ranged from 40 to 65. On the third day of hospitalization at the ICU (August 11), he received ceftriaxone and doxycycline for high white blood cell count and possible pneumonia due to aspiration.

During hospitalization in the ICU, he developed frequent ventricular premature beats, renal failure, hyperkalemia and acidosis. On 23 September 2017, his blood pressure and heart rate decreased progressively. He expired at 10:03 that day.

### 2.2. mNGS

#### 2.2.1. Sample processing and RNA extraction

According to the manufacturer’s protocol, total genomic DNA and RNA were extracted directly from 500 uL CSF (August 17) using QIAAMP VIRALRNA MINI KIT (52904#, QIAGEN) for extraction. Complementary DNA (cDNA) was generated from the RNA template by reverse transcription using SuperScript™ II Reverse Transcriptase (18064022, Invitrogen).

#### 2.2.2. Construction of DNA library and sequencing

The protocol for the construction of a DNA library and sequencing was reported previously (12). In brief, the DNA library was constructed through DNA-fragmentation, end-repair, adapter-ligation and PCR amplification. The constructed library was characterized by Agilent 2,100 (Agilent Technologies, Santa Clara, CA, USA) and Qubit 2.0 (Invitrogen, Waltham, MA, USA). The double strand DNA library was transformed into a single-stranded circular DNA library (13). DNA nanoballs (DNBs) were generated from single-stranded circular DNA using rolling circle amplification (RCA) (14). The DNBs were quantified using Qubit 2.0. DNBs were loaded on the flow cell and sequenced on the BGISEQ-100 sequencing platform (BGI-Tianjin, Tianjin, China) (12).

#### 2.2.3. mNGS analysis

High-quality raw data obtained during mNGS were generated by removing the low-quality sessions, followed by computational subtraction of human host sequences mapped to the human reference sequence (hg19) using Burrows–Wheeler Alignment (15). The remaining data were classified by simultaneously aligning to four Microbial Genome Databases, including viruses, bacteria, fungi, and parasites. The databases were downloaded from NCBI.^1^ Databases contain 4,152 whole genome sequences of viral taxa, 3,446 bacterial genomes or scaffolds, 206 fungi related to human infection, and 140 parasites associated with human diseases (16, 17). The number of unique alignment reads was calculated and standardized to get the number of reads stringently mapped to pathogen species and processed by the cloud-computing pipeline for metagenomic identification of pathogens (Supplementary material 1). A control sample from a non-infected patient was subjected to the aforementioned procedures.

### 2.3. Validation by TaqMan PCR and sequencing

When rabies was supported by the mNGS, we sent new saliva samples, which were collected into a sterile tube using a one-shot non-bacterium injector, to the local CDC laboratory and to a veterinary rabies research center for independent validation. Since the TaqMan PCR and sequencing for rabies were sent exclusively to the CDC and rabies research center, they were not undertaken by BGI on the same samples.

#### 2.3.1. TaqMan PCR

The TaqMan PCR was performed (August 29) according to the manufacture’s protocol (Aodonginspection & testing, Shenzhen, China). The reaction volume comprised 16 μl of nucleic acid amplification buffer (Tris, KCL, dNTPs, MgCL2), 2 μl of each primer and probe, 2 μl of enzyme mix (reverse transcriptase enzyme, RNA enzyme inhibitor TaqMan), and 5 μl of sample RNA. Samples were amplified using the following program settings: 1 cycle each of 50°C for 30 min, and then 95°C for 5 min, followed by 40 two-step cycles each of 95°C for 10s and 55°C for 40s. The primers and probes were provided in Supplementary material 2.

Positive and negative controls for rabies virus were performed simultaneously. The Ct value of positive control should be less than 35.0, and typical amplification curve should be evident to consider the experiment valid.

#### 2.3.2. RT-PCR and sequencing

Total RNA of saliva (August 29) was extracted individually with TRIzol (Invitrogen, Carlsbad, CA, USA). RT-PCR was carried out using a previously published protocol (18). The PCR products were purified and cloned into the pMD18-T vector (TaKaRa, Dalian, China). The sequencing of selected positively identified clones was done at least twice in both directions (Nanjing Genscript Biological Technology Co., Ltd., China). The primer sets for PCR amplification were based on those Lu et al. (19). The Primers were provided in Supplementary material 2.

### 2.4. Phylogenetic analysis

Sequences from the 250 bp fragment of RT-PCR and NGS were used for phylogenetic analysis. Multiple alignment of the above sequences was performed using CLUSTALW on partial gene sequences from GenBank. DNAstar 7.1 was used to determine sequence alignment. the MEGA7 package was used to construct phylogenetic trees using a maximum likelihood method with 1,000 bootstrap replicates.

### 2.5. Ethics statement

Serum and CSF samples were used for virologic analysis after obtaining written informed consent from the patient’s family. The clinical and laboratory data of the patient were sent to the corresponding author without personally identifying information. The research and ethics committees of the Ethics Committee of Shenzhen Hospital South Medical University approved the protocols and procedures (Approved No. of ethic committee: NYSZYYEC20190005).

## 3. Results

### 3.1. mNGS

After excluding reads of low quality and all human genomic sequences, the remaining reads were aligned to the RefSeq microbial genomic database. Two sequence reads uniquely aligned with the rabies virus (Table 1). The two reads were 99 and 97% homologous with genome of rabies virus (NCBI No. NC_001542.1), respectively.

**TABLE 1:** Sequence reads related to rabies virus by mNGS and RT-PCR from the patient.

	Sequence reads (5′→3′)
mNGS	GGGGCTGTCTATACTCGAATCATATGAATGGAGGTCGACTAAAGAGATCACATATAAGGAGATATGTTTCAGTCAGTTCCAATCATCAAGCCCGTC CAAACTCATTTGCTGAGTTTCTAAATAAAACGTATTCTAG GGGCTCGTCTATACTCGAATCATGATGAATGGAGGTCGACTAAAGAGATCCACATATAAGGAGATATGTTTCAGTCAGTTCCAATCATCAAGCCCGT CCAAACTCATTGCTGAGTTTCTAAATAAAACGTCATTCTACG
RT-PCR	CCGGAACTCGATCTTAGCTGGACCTACGACATGTTCTTCTCCCGGATTGAACATCTCTATTCAGCTATCAGAGTGGGTACTGTTGTCACTGCTTAC GAAGATTGCTCAGGACTGGTATCATTTACAGGGTTCATAAAGCAAATAAATCTCACTGCAAGAGAAGCAATACTATATTTCTTCCATAAGAAC TTTGAGGAAGAGATAAGAAGAATGTTCGAACCAGGGCTAAA

### 3.2. TaqMan PCR

A TaqMan PCR was performed on the CSF and saliva of the patient. The sample from saliva was positive for rabies virus with Ct value of 29.7, while the sample from CSF was negative (Supplementary material 3).

### 3.3. RT-PCR and Sanger sequencing

**FIGURE 1:**
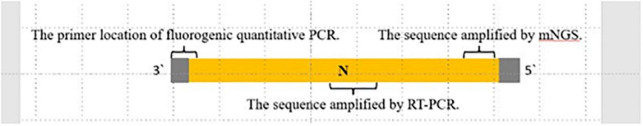
The location and length of sequences. Sequences were identified by mNGS, RT-PCR/Sanger sequencing, and quantitative RT-PCR. Shown in proportion to the whole rabies virus nucleoprotein N gene.

Rabies-specific PCR and Sanger sequencing obtained a 230 bp gene sequence from CSF (Table 1). The location and length of sequences identified by mNGS, RT-PCR/Sanger sequencing, and the TaqMan probe are shown in Figure 1.

### 3.4. Phylogenetic analysis

**FIGURE 2:**
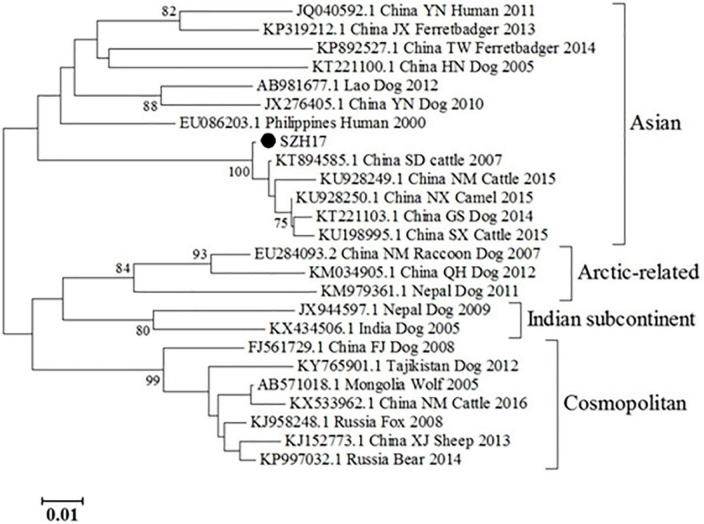
Phylogenetic analysis of RABVs. SZH17 was the RABV strain from the patient in this study.

The partial N gene sequence (230 bp) of the specimen was designated as SZH17. Sequence comparison showed that the N gene of SZH17 was 99.54% identical to SDJNC01, which was previously identified from a cattle rabies outbreak caused by a rabid dog in Shandong province, in 2007. SZH17 was grouped with the Asian clade (Figure 2), the most broadly distributed clade in China (20).

## 4. Discussion

In this study, we employed NGS and metagenomic data analysis to identify rabies virus in the CSF from a patient with rapidly progressive encephalitis without a convincing history of rabies exposure. Identification was confirmed by TaqMan PCR and Sanger sequencing in independent laboratories. These results indicate that mNGS can be a useful diagnostic tool for rabies in situations of low clinical uncertainty or when conventional rabies diagnostics are not available.

This patient had close contact with suspicious dogs within 1 week before the onset. An incubation period of just 5 days is extremely rare and led to diagnostic uncertainty. The patient maybe has attacked by another dog that transmitted rabies, which is hard to confirm or rule out. Although the patient presented with typical features of rabies, the diagnostic strategies for rabies that rely on epidemiology and clinical symptoms are not optimal for managing encephalomyelitis, which could be related to multiple infectious and non-infectious etiologies (21). Commercial PCR kits for rabies are not broadly available worldwide, including in China. Now survivors were occasionally reported in the literatures, an accurate laboratory-based diagnosis is essential. Through mNGS, we established a timely etiologic diagnosis for this patient without a convincing rabies exposure history of rabies. Theoretically, mNGS can sample without bias, and it facilitates the identification of pathogens, whether expected or unexpected, or even the discovery of new species. This technology is particularly advantageous when doctors fail to suspect pathogens (22). In a previous study, mNGS was used to identify rare, novel, or previously unconsidered pathogens in various clinical settings (23). For rabies patients without a clear exposure history, such as patients infected through organ transplantation and our patient, mNGS can help an accurate diagnosis.

It is not clear why only two sequences from the rabies virus were detected in the CSF sample, both from the nucleoprotein (N) gene. This may be explained by low abundance of rabies virions in CSF and the higher relative abundance of rabies virus N gene mRNA relative to later viral transcripts. Rabies virus is highly neurotropic, non-cytolytic, evades detection by low virus expression, and is transmitted trans-synaptically with exquisite precision, with budding of virions restricted to dendrites (24). Free rabies virus or virus mRNA is therefore infrequently detected by PCR or culture from cerebrospinal fluid (25). The nucleoprotein gene is the first gene to be transcribed in the single-stranded, negative sense, rabies virus genome structure, so is transcribed at higher levels than following genes (24).

Metagenomic next-generation sequencing may be superior to the traditional rabies tests, especially in non-epidemic areas. The current gold standard serological assay is the Rapid Fluorescent Focus Inhibition Test (RFFIT), which is recommended by both the Advisory Committee on Immunization Practices (ACIP), and the World Health Organization (WHO). However, it is not possible to make an early diagnosis (26). Biochemical methods based on PCR are not widely available in a non-epidemic area, such as Shenzhen which embraces an annual incidence of less than 1 per year (27, 28). Additionally, such methods are costly, both in time and financially, when there is no direction of diagnosis. mNGS can identify infectious agents in a target-independent manner. Thus, with mNGS, it is possible to achieve diagnosis in an early stage necessary for proper ICU care, and will identify diseases without clinical suspicion or when special diagnosis kits are unavailable (29). However, when the suspicion for rabies is high, it is desirable to choose a PCR-based molecular assay over mNGS.

Since the mNGS is a new diagnostic tool and not recommended in rabies-related guidelines, confirmation by other traditional assays is necessary. In this instance, we carried out specific TaqMan PCR and RT-PCR/Sanger sequencing of follow up saliva samples at outside laboratories to confirm the preliminary diagnosis by mNGS. The sequences by mNGS and sequencing were in different regions of the N gene, eliminating the chance of shared contamination (Figure 1). Phylogenetic analysis showed that the N gene of SZH17 was 99.54% identical with SDJNC01, which is broadly distributed and leads to more than half of animal and human rabies cases in China (20).

In our case, the TagMan PCR test was negative for CSF samples, while the RT-PCR test was positive. The possible reason for this phenomenon could be that the TagMan PCR and RT-PCR assay kits are from different manufacturers. The sensitivity may differs significantly due to the different target genes and Differences in manufacturing processes.

It is worth mentioning that the limitations of mNGS include contamination with reagents-derived nucleic acids, common index mis-assignment (23, 30), higher cost, equipment, personnel training, standardization of methodologies, data analysis, diagnostic accreditation, and methods to determine clinical relevance along with interpretation guidelines for clinicians (31). As with any new technology, the clinical application of mNGS will take some time as providers and users gain experience and mastery. These limitations will be overcome, and new guidelines for this technology will arrive.

In conclusion, we present preliminary evidence that mNGS may be considered a useful screening tool for rabies, especially when routine laboratory kits for rabies are not available or patients do not have a convincing exposure history. Despite its advantages, several barriers need to be addressed before regular implementation.

## Data availability statement

The original contributions presented in this study are included in this article/Supplementary material, further inquiries can be directed to the corresponding author.

## Ethics statement

This study was approved by the Ethics Committee of Shenzhen Hospital South Medical University (IRB No: NYSZYYEC20190011). The use of patients’ clinical data and CSF samples with de-identification for this study was approved by the Ethics Committee of Shenzhen Hospital South Medical University.

## Author contributions

JP and YL: conception and design of the study. YF, XW, YW, and XL: acquisition of data, analysis, and interpretation of data. YL, XM, RW, JG, and JT: drafting the article or revising it critically for important intellectual content. All authors contributed to the final approval of the version to be submitted.

## Footnotes

1
https://ftp.ncbi.nlm.nih.gov/genomes/
