# Supplementary material for: Metagenomic next-generation sequencing for the etiological diagnosis of rabies virus in cerebrospinal fluid
Source: Front Med (Lausanne). 2023 Feb 9;10:982290. doi: 10.3389/fmed.2023.982290 (PMC9947348; doi:10.3389/fmed.2023.982290)
Supplement: Supplementary file 1 [file Data_Sheet_1.PDF]

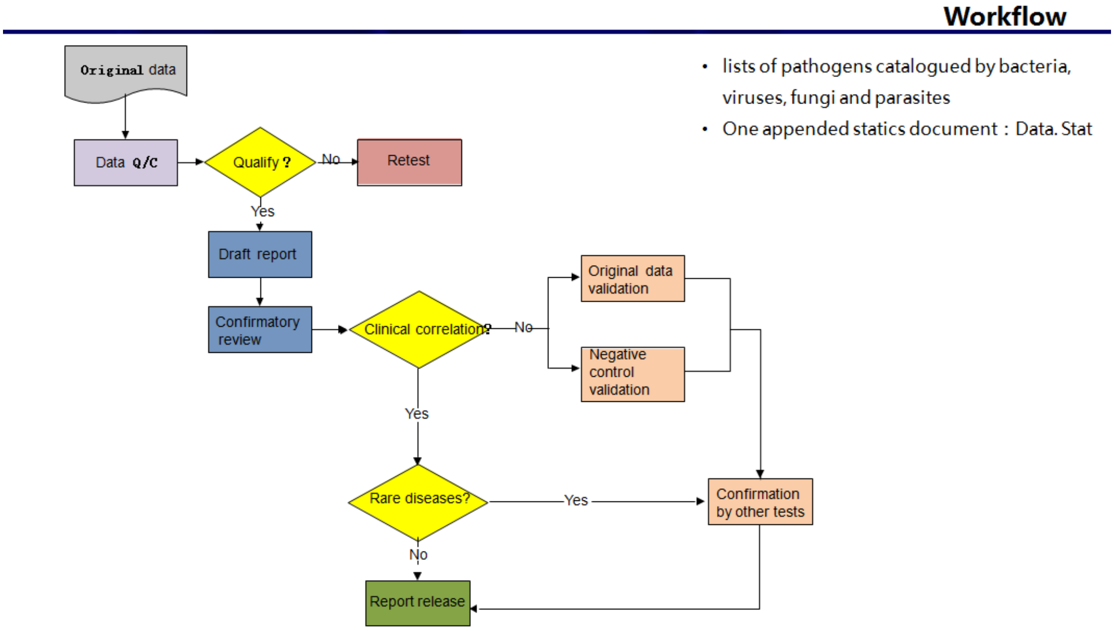

S1: The cloud-computing pipeline for metagenomic identification pathogens

Sequence reads were classified according to their origin by a bioinformatics pipeline. The results include four lists of detected bacteria, viruses, fungi and parasites respectively and one statistical document. Then these results were further interpreted for generation of clinically actionable reports by the following steps.

1) Quality control (QC) : Sequencing data output and ratio of host reads were calculated and examined to ensure that the data of microbes was enough for analysis; if the microbial data was less than the standard of care , resequencing would be arranged;

2) Report examination: If the data aligned to microbes was enough for QC, the four lists of different microbes and negative control would be examined to generate a interpretive report. Irrelevant information should be filtered, such as background microbes introduced in the sample collection;

3) Report confirmation: Before the report reaches the hands of the physician, the listed information would be confirmed to avoid any inconformity and error.
