# Supplementary material for: Metagenomic next-generation sequencing for the etiological diagnosis of rabies virus in cerebrospinal fluid
Source: Front Med (Lausanne). 2023 Feb 9;10:982290. doi: 10.3389/fmed.2023.982290 (PMC9947348; doi:10.3389/fmed.2023.982290)
Supplement: Supplementary file 2 [file Data_Sheet_2.PDF]

S2. The primers of TaqMan PCR and RT-PCR.

Table 1. The Primers and probes of TaqMan PCR

Name	Sequence
**Sense primer**	5'-CACGCTTAACAACAAAATCAGAGAA-3'
**Antisense primer**	5'-CCACCTGATTATTGACTTTGAAYACAA -3'
**Fluorescence probe**	5'-CTTGTCGGCATCCATTGTAGGGGTGT -3'(the 5' end of the probe is marked with a fluorescence emission group FAM, the 3' end was marked with a fluorescence quenching group TAMRA)

Table 2. The primers of RT-PCR

Name	Sequence	Position in N gene	Size
**NF2**	CAAGATGTGYGCYAAYTGGAG	644-899	255
**NR2**	AGCCCTGGTTCGA ACATTCT		

The RT polymerase chain was performed targeting at the conserved regions of the nucleoprotein gene N.
