# Supplementary material for: Metagenomic next-generation sequencing for the etiological diagnosis of rabies virus in cerebrospinal fluid
Source: Front Med (Lausanne). 2023 Feb 9;10:982290. doi: 10.3389/fmed.2023.982290 (PMC9947348; doi:10.3389/fmed.2023.982290)
Supplement: Supplementary file 3 [file Data_Sheet_3.PDF]

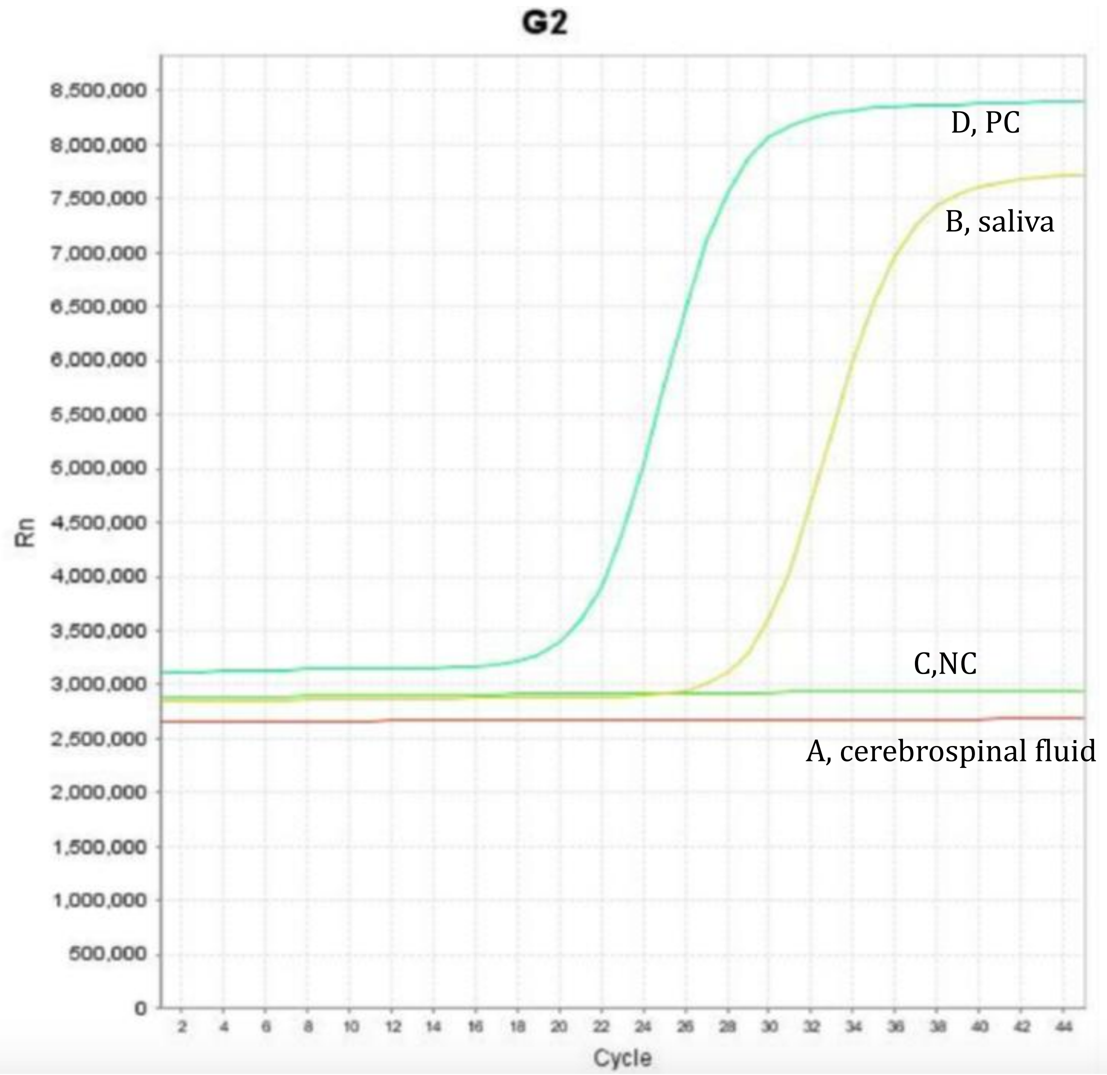

Well	Sample	Target	Task	Qty	Cт	Cт Mean	Cт SD	Qty Mean	Qty SD	Cт Threshold	Baseline Start	Baseline End
A6	Cerebrospinal fluid	G2	U		UND.					603,858.250	3	4
B6	Saliva	G2	U	0.000	29.709	29.709		0.000		603,858.250	3	21
C6	NC	G2	N		UND.					603,858.250	3	4
D6	PC	G2	S	1.000	21.650	21.650				603,858.250	3	14

S2. Plot of TaqMan-PCR analysis of Rabies virus amplification.

Cerebrospinal fluid (A), saliva (B), negative control (C), and positive control (D). NC = negative control, PC = positive control, S=Standard, N=NTC, U=Unknown, UND. = Undetermined.
